# Regional cooperation is essential to combatting health emergencies in the Global South

**DOI:** 10.1186/s12992-021-00659-7

**Published:** 2021-01-09

**Authors:** Ana B. Amaya, Philippe De Lombaerde

**Affiliations:** 1 College of Health Professions, Pace University, 163 William Street, New York, NY 10038 USA; 2 United Nations University Institute on Comparative Regional Integration Studies (UNU-CRIS), Potterierei 72, 8000 Brugge, Belgium; 3 NEOMA Business School, 1 Rue du Maréchal Juin, 76130 Mont-Saint-Aignan, Rouen, France

**Keywords:** Regional integration, Health policy, Global south, Low- and middle-income countries

## Abstract

Since COVID-19 was first discovered, it exploded into a pandemic resulting in devastating effects on human lives and a global recession. While there have been discussions that COVID-19 will accelerate the ‘end of globalization and multilateralism’, we have already seen the high costs of non-cooperation in responding to the virus resulting in sub-optimal use of resources, rapid spread of the virus between countries, and, ultimately, significant loss of life. In spite of their favorable demographic structures and relatively young populations, countries in the Global South are still harshly affected in both epidemiological and economic terms. Nations must find innovative ways to address health concerns and regional bodies are possible mechanisms for facilitating international cooperation on health. We delineate how regional organizations can support how countries address health threats namely by serving as a bridge between the global and national policy levels; strengthening disease surveillance; mobilizing supply chains and facilitating trade; supporting the production and procurement of medicines and supplies; and coordinating policies and work with other actors. We finalize by arguing that mechanisms for regional cooperation must be strengthened themselves in order to effectively contribute to positive health outcomes within member states.

## Background

While there have been discussions that as a result of COVID-19 countries will turn more inwardly, collaborate less with global institutions, and become more nationalistic, this is not a realistic option for many low- and middle- income countries. It is not a choice for many small and medium-sized countries either. Unfortunately, we have seen evidence of this thinking with the reemergence of ‘vaccine nationalism’. This is a phenomenon that was first evident during the H1N1 epidemic and recently we have observed it in practice when high-income countries have brokered deals with major pharmaceutical companies to preorder their still-in-development COVID-19 vaccines. Through these actions, countries prioritize their citizens’ self-interest in lieu of cooperating with other nations to find solutions. In the case of the USA this has also resulted in the country’s refusal to join the COVAX facility that aims to ensure more equitable distribution of vaccines between countries regardless of their ability to pay. Indeed, evidence shows that over 80% of the supply of one of the most promising vaccines with an efficacy rate of over 90% developed by Pfizer, has already been claimed by the United States (US), United Kingdom (UK), European Union (EU), Canada and Japan [1]. Even if the vaccine were to be made available at a feasible rate to some low- and middle- income countries, they would also need to grapple with the high costs of maintaining, storing and transporting the vaccine, as well as deploying it among their citizens.

Amidst high numbers of cases in Latin America, Southeast Asia, India, and South Africa and evidence that COVID-19 has impacted countries in the Global South harshly in epidemiological and economic terms [2] and will continue to do so if there are resurgent waves of the disease, these countries must find innovative ways to address their national health concerns and regional cooperation is one of the mechanisms to do so.

Regional cooperation between neighboring countries refers to a wide variety of forms of collaboration, ranging from informal cooperation, setting up joint projects (e.g. to build common infrastructure), coordinating policies and regulatory frameworks, to shaping joint policies and institutions. If the latter is the case, we refer to regional organizations. Regional organizations can be supranational organizations (like in the case of the EU) or inter-governmental organizations, where countries do not surrender power to the wider organization, which is the most common form of cooperation (e.g. in the Association of Southeast Asian Nations -ASEAN-; Southern Common Market – Mercosur-, or the Southern African Development Community – SADC-). These organizations emerged from recognizing that neighboring countries share common characteristics, in many cases sharing similar histories and cultures; geographic features; and – especially - shared problems. These shared problems may comprise of governance challenges and/or geo-political and economic interests. However, as we will see later on, a particular regional institutional design or cooperation modality does not fully define the functions of an organization.

While the past decades have been characterized by an increased participation of regional organizations in the Global South on social issues, their effectiveness has been constrained by not only existing inequities in power and influence within its member states; but also inequities at the international level. As a result of this, other high-income nations or blocs (e.g. the EU) have been able to shape global health policy in more effective ways. In addition, regional organizations do not exist in a void and must contend with their commitments to other alliances (given that countries frequently belong to different groupings or blocs); prevailing tensions within countries between protecting national interests versus building shared goals for social issues, such as health; as well as the local politics of its members. Indeed, in the case of the now defunct Union of South American Nations (UNASUR), its country support disintegrated as soon as there were political shifts in the region. Paradoxically before UNASUR experienced its decline, it was known for its relatively effective regional cooperation in health policy. Furthermore, similar to other international institutions, inter-governmental regional organizations serve the member states and not vice versa, meaning these institutions have little to no power to hold member states accountable to their commitments and most policymaking occurs through consensus, severely hampering explicit actions on health.

Importantly, it would be simplistic to suggest that we can identify clear links between the level of institutionalization of a regional organization and the importance or effectiveness of regional health policies. For instance in the case of the EU, which is a heavily institutionalized regional organization, primary responsibilities regarding health policies still lie with the member states. This may in part explain the perceived lack of a united EU response to the COVID-19 crisis. In addition, complementary European health policies have only gradually been introduced by integrating them in the context of the regulation of the internal market. In the case of the UNASUR, it has been argued that the relative success of their involvement in health was not so much the result of creating heavy institutions or producing regional health rules, but rather stemmed from creating light sector-specific mechanisms capable of reducing transaction costs, promoting information sharing and serving as catalysts for the diffusion of best practices and cross-country cooperation [3].

Ultimately, the question is whether regional organizations, or regional arrangements more generally, can be effective when faced with cross-border policy challenges such as COVID-19.

## How can regional organizations support their member states during health emergencies?

We know that regional cooperation works in health emergencies. An outbreak of another novel virus in 2002, Severe Acute Respiratory Syndrome (SARS), not only led to revised International Health Regulations, but it also showed how regional cooperation in health can work in modern times to address emergency situations. Following an understanding that countries could not control SARS on their own, ASEAN convened a meeting at the time to develop practical advice to support countries’ responses as well as strict measures to contain the spread throughout the continent [4]. ASEAN’s response was lauded by the World Health Organization (WHO) as an example of effective international cooperation against a common enemy, contributing to the minimal spread of the disease around the world [5].

Despite the lack of research on the topic, what is clear is that in practice the current COVID-19 situation demonstrates the cost of non-cooperation. We have seen uncoordinated quarantine measures; lockdown measures and border closures (leading to congestions in certain border cities); unorganized repatriation of foreign nationals from several countries; uncoordinated measures to control and facilitate the trade of medical supplies; among other issues. The lack of coordination in all these cases has led to either unnecessarily spreading the virus to neighboring countries and/or a sub-optimal use of medical supplies and infrastructure.

We argue that regional cooperation, in general, and regional organizations, in particular, can support countries in the current crisis as well as help minimize the risks related to subsequent waves of COVID-19. This is even more critical in the case of countries in the Global South which do not have the same resources, not only in terms of hospital capacity, testing supplies, and the number and distribution of health care workers; but also in terms of economic sustainability compared with the Global North.

### Areas for regional cooperation to respond to the COVID-19 crisis

As already discussed, it is not possible to put all regional organizations in one box; we know they vary in composition, institutionalization and approach to health [6]. However, opportunities exist for collaboration either as formalized institutions or ad hoc ones created to address the pandemic. Below we describe some examples of areas where regional groupings can act to address COVID-19 or other health emergencies.

#### Serving as a bridge between the global and national level

In our research we have found how regional organizations can serve dual roles while acting as intermediaries between the global and national levels. These bodies can help their member states in a vertical manner, for instance by translating agreements and guidelines from the global level (e.g. the Sustainable Development Goals) to national policies and targets appropriate to their specific settings and mobilizing resources to reach these goals; as well as horizontally with the countries by providing data and support to address cross-border policy challenges and supporting evidence-based coordination of these goals [7]. At the same time, regional organizations can coordinate their responses with the WHO regional offices (such as the Pan American Health Organization [PAHO], South-East Asia Region [SEARO], and Regional Office for Africa [AFRO]) following their technical guidance, as well as supporting epidemiological surveillance and case identification by encouraging data sharing across countries. For example, as we will discuss later, following a proposal by Costa Rica, PAHO worked with its member states to share knowledge and access to COVID-19 therapeutics and vaccines through the COVID-19 Technology Access Pool (C-TAP) initiative. Regional bodies have also historically collaborated with WHO regional offices, following their technical guidelines and developing action plans based on their input. However, the differences between both types of institutions are clear. Whereas WHO regional offices are usually agencies based on continental groupings (at least in the cases of PAHO and AFRO), regional organizations involved in health are guided by the unique needs of their member states with specific health concerns and are likely to take more normative positions for health. WHO regional offices are unable to do so given their composition and mandate. For instance, UNASUR clearly took the charge to advance the ideal of health as a human right in international discussions and forums, which is something that PAHO could not do as forcefully. This means regional organizations can be more courageous in proposing systemic change on how health issues are addressed since they are not constrained by a mandate as technical agencies that are apolitical and as we know, as much as we try to avoid it, health policies and decision-making are innately political. Due to this, groupings of countries guided by regional organizations, for example, can take stronger stances to guide the work of WHO regional offices if they have enough convening power to do so, thus tipping the scales within an international organization. We have seen increasing signs of this in the case of the WHO, which has traditionally been constrained by the budgetary support of high-income countries, with several African countries recently calling for a redistribution in how the WHO budget is allocated between the WHO headquarters’ office and regional agencies.

Regional bodies can also advocate for their member states by developing joint positions in international forums such as the World Health Assembly to obtain support for their policy goals and negotiate with other nations or blocs on key issues that impact low- and middle- income countries. Given the importance of a vaccine to address COVID-19, the COVAX facility, a mechanism to pool the procurement and distribution of vaccines, has been a welcome partnership among several countries. However, this initiative has been criticized for issues such as lack of transparency on the vaccine strategy, price and possible risks. Moreover, questions still remain on whether pharmaceutical companies can be held liable under this agreement over possible deaths or side effects [8]. Ultimately, initiatives such as these should avoid reproducing past mistakes in inequitable decision-making that have persisted over time in global policy, by giving a voice to low- and middle- income countries participating in the scheme. Regional organizations have the potential to bring out these concerns and negotiate on the basis of strength in numbers with powerful pharmaceutical companies.

#### Facilitating cross-border mobilization of goods

The COVID-19 pandemic demonstrated how ill-prepared countries were on many levels but perhaps most visible was the lack of supplies in clinical settings, in terms of ventilators but also swabs and testing kits, among other supplies. This was compounded in many cases by closures of borders between countries, which left many citizens stranded in neighboring countries and limited trade between areas. Apart from ‘constitutionalizing’ intra-regional free trade by means of free trade areas, customs unions or common markets, regional organizations can play an important role in supporting the mobilization of supply chains and facilitating the transportation of critical medical supplies by implementing inter-state agreements. For example, early on in the pandemic, SADC adopted guidelines to facilitate the movement of essential goods across borders while limiting the spread of the disease [9]. Regional groupings should consider similar actions in future health emergencies, as well as working together to coordinate border closures and quarantine measures if necessary.

Even if it might not be realistic or even desirable to regionally centralize or pool strategic medical supplies, regional management mechanisms might make a lot of sense so that a temporary overcapacity in one member state can be matched with lower capacity in another member state. Likewise, even in conditions of lockdown, mutual and regulated cross-border access to hospital capacity in neighboring border cities might save lives. This was illustrated by Germany, Austria and Luxembourg accepting COVID-19 patients from Italy, France and The Netherlands during the first wave of COVID-19 [10].

#### Pooling resources for the production and procurement of medicines and supplies

Based on past experience, countries like India and Brazil have demonstrated ability to produce therapeutics or vaccines for COVID-19; however, a large number of countries in the Global South do not have the technology, patents or facilities to produce medications and supplies such as masks. India and South Africa submitted a joint proposal for the Trade-Related Aspects of Intellectual Property Rights (TRIPS) Council to recommend the World Trade Organization grant a waiver from some obligations under the TRIPS agreement to avoid barriers to scaling up health technologies to respond to the COVID-19 pandemic, highlighting the need for rapid access to affordable medical products, personal protective equipment as well as medicines and vaccines. Given its significance, these proposals were supported by several other WTO members from the Global South but opposed by some high-income countries [11]. While this proposal has been supported by several intergovernmental and non-governmental organizations, including the African Union (AU) [12]; regional organizations from the Global South still haven’t fully exploited their convening power in these forums nor do they have the same experience and capacity to negotiate these agreements compared to the EU and other high-income countries. While significant efforts were made by institutions such as UNASUR and SADC to strengthen the training of their diplomats in health issues, this is an ongoing process that requires more investment and attention.

At the same time, regional organizations can facilitate the joint procurement of essential medicines, medical supplies and other equipment through pooled purchasing. This not only ensures a lower price through bulk purchasing but also equalizes the negotiating power of countries, especially lower-income countries, whom otherwise would not be competitive. Mercosur started experimenting with this in 2015 in collaboration with the PAHO Strategic Fund in order to jointly procure high-cost medicines, initially to treat hepatitis C and HIV/AIDS, resulting in cost savings of around US$20 million [13].

Several regional efforts have already emerged to respond to COVID-19. Following an initiative by Costa Rica, PAHO proposed a technology platform named (C-TAP) to facilitate the equitable sharing of knowledge, data and intellectual property on COVID-19, as well as access to effective vaccines, medicines and other health products [14]. In addition, several regional grouping such as Mercosur and the South Asian Association for Regional Cooperation (SAARC) have also approved emergency funds to support their member states during the COVID-19 crisis [15].

#### Coordinating actions with donors and external partners

Low and middle countries already face capacity constraints to address their own domestic issues. While external support is welcome, it frequently has unintended consequences and costs related to communicating and reporting to different donors with various requirements (what is commonly referred to as transaction costs), leading to negative effects on already limited country capacities. Regional organizations can coordinate the work of donors and partners to support countries. For example, the (AU) coordinated the donation of medical supplies from the Jack Ma Foundation to its member states consisting of surgical supplies, ventilators, protective clothing, etc. [16]. Countries can also begin to set the basis for their recovery by generating mechanisms to reduce the economic and social effects of the pandemic by jointly negotiating debt forgiveness from multi-lateral banks and partnering with donors for regional growth, for example. They can also support countries mobilize resources for other existing health initiatives or concurrent health emergencies, such as maternal and child health programs or outbreaks of diseases such as Ebola or Dengue fever, which may otherwise experience a shortfall in funding due to the focus on COVID-19.

## Conclusion

Regional organizations vary from relatively light intergovernmental coordination mechanisms to comparably heavy supranational institutions. Independent of their particular institutional characteristics, they have the potential to play a role in addressing health emergencies especially with respect to coordinating mobility measures for citizens, protecting cross-border flows of medical and food supplies, jointly procuring medicines and strategically managing resources, among other actions.

It is clear that countries within each region have particular needs and resources, as well as different degrees of vulnerability to epidemics and thus must deal with their own complexities that require local and specific solutions. While regional cooperation is not the silver bullet that will solve the problems caused by COVID-19, it can be part of the solution particularly in the Global South. For this to happen, mechanisms for cooperation must be strengthened themselves. In some cases, this will require managing the short-term self-interests of countries and encouraging binding resolutions to avoid inaction. It will also require political pragmatism and solutions adapted to specific regional contexts. The COVID-19 crisis has already shown us what the costs of non-cooperation are, now it is time to leverage existing structures to control the current pandemic and – even more importantly - to better prepare for the next one.

## Data Availability

Not applicable.
